# The responsibility of thwarting and managing Japanese encephalitis cannot be understated—Outbreaks or resurgences may manifest, catching us off guard

**DOI:** 10.1371/journal.pntd.0011698

**Published:** 2023-11-02

**Authors:** Ran Wang, Zhengde Xie

**Affiliations:** 1 Beijing Key Laboratory of Pediatric Respiratory Infectious Diseases, Key Laboratory of Major Diseases in Children, Ministry of Education, National Clinical Research Center for Respiratory Diseases, Laboratory of Infection and Virology, Beijing Pediatric Research Institute, Beijing Children’s Hospital, Capital Medical University, National Center for Children’s Health, Beijing, China; 2 Research Unit of Critical Infection in Children, 2019RU016, Chinese Academy of Medical Sciences, Beijing, China; Universita degli Studi di Pavia, ITALY

Japanese encephalitis virus (JEV) is a flavivirus with a single-stranded positive RNA genome mainly distributed in Asia and the Western Pacific. By 2022, it had spread to the south of Cairns, Australia. Japanese encephalitis (JE) is an acute zoonotic infectious disease mainly transmitted through mosquito bites, particularly in rural areas and during seasonal peak disease periods. It is mainly characterized by central nervous system diseases and is associated with acute onset, severe illness, high disability rate, and a heavy disease burden. Some severe patients have left serious neurological sequelae [1]. There is no specific treatment for JE at present, and mosquito control has failed to eliminate the disease. Fortunately, JE is a vaccine-preventable disease, and vaccination remains a critical means of preventing and controlling JE [2]. Since the implementation of the planned immunization of JE vaccine in 2008, the incidence rate of JE in children has significantly reduced in China, where the disease has been prevalent for a long time. However, despite the effective vaccines, JE remains a grave health threat in numerous underdeveloped countries and regions due to the lack of good medical equipment and human resources, as well as the relatively weak public awareness of disease prevention. For example, among all etiological factors of flavivirus infections, JE causes the highest hospitalization rate and the most serious disease burden among Chinese children [3]. Additionally, adult cases are also exhibiting a gradual increase [4], underscoring the ongoing relevance of JE as a potential public health concern, which poses a substantial threat to the well-being of the population.

In recent years, the prevention and control of the JE epidemic has encountered new situations and challenges. Firstly, the spread of JEV has extended to nontraditional epidemic areas [5]. With a high variability, strong adaptability, diverse storage, and transmission hosts, the virus can spread and circulate among animals for long period, making it challenging to eliminate. Climate change, as well as frequent human movement, further increases the transmission risk and extends the monitoring area and objects. Secondly, JEV genotypes coexist and switch, and the risk of emergence and reemergence of JEV genotype V increases. Based on envelope protein nucleotide sequence differences, JEV is categorized into 5 genotypes (GI to GV), each with its geographical distribution characteristics. Certain genotypes of JEV are capable of generating elevated viremia levels with prolonged durations in ducklings and chicks, coupled with diminished interferon (IFN) production [6,7], making the virus better suited for bird hosts. The current approved mainstream JE vaccines are based on GIII [8]. However, the switching of dominant epidemic genotypes has occurred, such as China’s conversion from GIII to GI in the 2000s [9] and Korea’s recent conversion to GV [10]. The RNA coding and noncoding regions of GV JEV have more mutation sites than the other 4 genotypes, which makes it highly distinctive and pathogenic [11]. These prevailing JEV strains may consist of one or multiple genotypes, and it is necessary to establish sensitive and reliable detection methods to identify various genotypes. Additionally, the prevalence of multiple JEV genotypes poses challenges to the effectiveness of current vaccines developed based on GIII. For instance, immunization using the live attenuated vaccine SA14-14-2, which is based on the GIII, remains effective in preventing diseases caused by GIII and GI, but there may be a weakening in its protection against GV [12,13]. Thirdly, the enhancement effect mediated by cross-reactive antibodies in heterologous flavivirus infection should be considered. Antibodies generated by JE vaccination have been shown to have an antibody-dependent enhancement effect on dengue virus (DENV) or Zika virus (ZIKV) infections [14,15]. Nonetheless, it is crucial to recognize that these findings are presently confined to observations in vitro or within animal experiments. In real-world situations, no reported data have established a connection between the administration of JE vaccines and an elevated risk of severe DENV and ZIKV infections. However, drawing from the experience in designing dengue and Zika vaccines that effectively bypass the antibody-dependent enhancement (ADE) effect, it becomes evident that confronting and overcoming this ADE challenge is a challenge that cannot be ignored in the development of novel JE vaccines. Fourthly, JEV is an RNA virus that, when exposed to a myriad of factors, including natural selection pressure, possesses the capability to undergo mutations. These mutations can ultimately lead to immune escape, resulting in a decrease in the effectiveness of vaccines. Therefore, the design strategy of the vaccine must conform to the current epidemic status and trend. Fifthly, the significance of T cell involvement in JEV infection protection warrants thorough consideration. Individuals who have experienced JEV infection exhibit an IFNγ response from both their CD8^+^ and CD4^+^ T cells [16]. Moreover, despite the high homology of antigen sequences that trigger T cell immunity among flaviviruses, there are divergent perspectives on the extent of cross-reactive T cell responses [16,17]. Consequently, it remains uncertain whether T cell immunity induced by heterologous flaviviruses can confer cross-reactive T cell immunity against JEV. Additionally, in a study involving 60 JEV vaccine recipients aged 19 to 20, even though they retained a certain level of neutralizing antibodies, discerning significant JEV-specific T cell responses proved undetectable [18], this suggests that the duration of T cell–mediated immunity may be shorter than that of humoral immunity. While it remains uncertain whether this is correlated with the rise in adult JE cases, it underscores the pivotal role of T cells in protective immunity, which should not be underestimated. In the development of vaccines, it is crucial to give due consideration to the induction of enduring JEV-specific T cell immunity, even against multiple genotypes.

Overall, in addition to maintaining current prevention and control policies, including scheduled immunizations and traveler vaccinations, it is also crucial to prioritize the strategic development of a new JE vaccine as a resource reserve to confront the novel situations and challenges that arise in the field of prevention and control. While this viewpoint primarily delves into the pivotal role of vaccines in the prevention and management of JE, it’s crucial to recognize that in regions such as Japan, Singapore, and Hong Kong SAR, where JE is relatively well managed, the reliance solely on vaccination may not be the sole strategy. Physical prevention and ecological management also wield significant influence, collectively shaping a comprehensive approach to JE prevention and control. Effective vector control, particularly in managing mosquitoes, emerges as a vital component. Practices like employing mosquito nets, donning long-sleeved clothing and trousers to minimize skin exposure, and using mosquito repellents all contribute to curtailing the risk of JEV transmission. Moreover, the vigilant supervision of ponds, lakes, and water bodies aids in reducing potential mosquito breeding sites. Considering that pigs are deemed intermediate hosts for JEV, their role in the ecosystem is noteworthy. JEV can find its way into the environment through contaminated pig excreta. Also, the hygienic conditions of pig farms and other agricultural operations significantly impact mosquito populations. These areas may not only serve as breeding grounds for mosquitoes but also, when located near residential zones, elevate the likelihood of human–animal interactions. Consequently, a comprehensive approach encompassing vaccination, vector control, ecological management, and hygiene practices is essential for effective JE prevention and control, particularly in high-risk regions. Ultimately, being fully prepared for an epidemic is essential, as it is better to anticipate potential threats than to react to them after the fact.
